# Introduction for bioinspiration

**DOI:** 10.1098/rsfs.2015.0052

**Published:** 2015-08-06

**Authors:** Denis Noble, Clemens Kaminski

**Affiliations:** 1 Editor, *Interface Focus*; 2 Department of Physiology, University of Oxford, Parks Road, Oxford, UK; 3 Department of Chemical Engineering and Biotechnology, University Cambridge, Pembroke Street, Cambridge, UK

The articles published in this issue of *Interface Focus* form a very special contribution to the celebrations during 2015 of the 350th anniversary of the first scientific journal, *Philosophical Transactions of the Royal Society*. We are delighted that the Society chose to publish the articles in one of the very newest journals of the Society. *Interface Focus*, similar to its parent journal *Interface*, echoes the original interdisciplinary nature of *Philosophical Transactions* before it was divided into the A and B series. We really do span across the whole breadth of physical and biological disciplines.

This issue on bioinspiration illustrates that breadth in examining the way in which the structures and functions of living organisms have inspired new forms of technology. Biological structures and processes have benefited from billions of years of refinement during the process of the evolution of life on Earth. So why should we not benefit from what nature discovered in designing our own new technologies? This meeting brought together scientists from a range of disciplines to discuss how nature has inspired their work and how this can contribute to the technological evolution.

The collection of papers begins with an engaging review by Whitesides [1] on the topic of *biomimetics*. He outlines clearly what is to be gained from mimicking nature's processes and materials, and that ‘bioinspiration’ spurs whole new branches of chemical research.

Deng & Almsherqi [2] look at a remarkable microbe that is able to survive in extreme environments, such as lack of food, thermal and osmolarity fluctuations, and high levels of reactive oxygen species. The way in which this is achieved through the ‘crystallization’ of the membrane into cubic structures provides clues to how phospholipids may not only facilitate these structural characteristics in such extreme conditions, but also provide a protective shelter for RNAs. It is not yet clear what insights this may provide for biotechnology, but it certainly provides insights into the evolution of life on Earth and how it survived harsh conditions.

In an article with the intriguing title ‘Crystals: animal, vegetable or mineral?’, Hyde [3] explores the evolution of our understanding of biomolecules that can assemble into aperiodic structures *in vivo*, so enabling us to understand structures across a wide spectrum of materials, from living to inanimate, with particular focus on the development of crystallography in materials science and biology.

Lagny & Bassereau [4] show how biologists have worked for a long time on deciphering how membranes are organized, and how they contribute to trafficking, motility, cytokinesis, cell–cell communication, information transport, etc., using top-down approaches and ever more advanced techniques. They ask and answer the question ‘what can we still do with synthetic systems, where do we stop building-up and which are the alternative solutions?’.

Cutkosky [5] shows how we can go in the reverse direction: from bioinspiration to biounderstanding, to show that the process of extracting principles from animals and adapting them to robots provides insights for both robotics and biology.

Schaber *et al.* [6] have contributed an article on modelling clustering of vertically aligned nanotube arrays (VACNT) to show how such modelling can inspire the adaption of VACNT arrays for biomimetic applications.

Patole *et al.* [7] take inspiration from photosynthetic systems in nature. Nanofabrication methods are described that lead to the self-assembly of light-harvesting proteins into patterned monolayers that may one day enable the storage of energy.

Hollfelder *et al.* [8] describes the use of small droplet compartments, generated in microfluidic devices, to mimic genetic evolution and how this bioinspired technology may lead to the discovery efficient molecular therapeutics.

Goldstein & van de Meent [9] consider ‘life at small Reynolds numbers'. Fluid transport in nature's building blocks, cells, is almost universally governed by diffusion because of the small prevailing length scales. There are fascinating exceptions, however: he shows that convective phenomena take place in certain giant cells types and that these may confer functional advantages.

The meeting at which the papers were presented was a great success. The oral presentations and discussions raised many new and exciting directions for research. The 350th anniversary celebrations offered a timely occasion for the Royal Society to explore a new ways of disseminating science to the wider world: the meeting was broadcast live and attended in satellite meetings in India and Brazil and there was active participation of online viewers during the discussion sessions. Recordings of every session can be viewed at http://rsfs.royalsocietypublishing.org/content/bioinspiration-new-technologies.
